# Letter from John Mortimer, Esq. Late Surgeon of His Majesty's Naval Hospitals at Jamaica, Barbadoes, Antigua, &c. to Dr. Johnson, Inclosing a Copy of an Official Report to the Commissioners of Transports, &c. Relative to the Yellow or Bulam Fever

**Published:** 1816-12

**Authors:** John Mortimer


### JOURNAL AND REVIEW.

VOL. II.] DECEMBER, 1816. [NO. 12.

### PART I.

### ORIGINAL COMMUNICATIONS.

—— quid utile ——

*Letter from John Mortimer, Esq. late Surgeon of His Majesty's Naval Hospitals at Jamaica, Barbadoes, Antigua, &c. to Dr. Johnson, inclosing a Copy of an official Report to the Commissioners of Transports, &c. relative to the Yellow or Bulam Fever.*

DEAR SIR, October 3d, 1816.

ONE of the objects I had in view in drawing up the paper herewith sent you for your perusal was, to shew that the fever, which of late years has occasionally appeared in the West Indies as an epidemic, is the same as that Dr. Pym saw at Gibraltar and at Cadiz—is the same that Dr. Rush wrote on; first, to prove its foreign origin and contagious influence; and in a second publication, when his judgement had been more matured, to acknowledge the errors of the first by avowing the contrary, but correct opinion.

Subsequent opportunities of seeing this Protean disease in different quarters of the globe, in all its various approaches, as well as the characters presented by it, in its progress to recovery or death, had (I had thought) left no doubt in the minds of the medical world, of its being, in every instance, of local origin; that though the constitution (bearing always in recollection the greater or less predisposition of individuals, dependent, perhaps, on age, temperament, and seasoning,) long exposed to the influence of such localities, very frequently became subject to its effects; yet, that but one opinion pervaded the medical corps, of the incapability of a second person contracting the disease from the first, attacked as above stated.

You may suppose how much surprised I was, on receiving a book, written by Mr. now Dr. Pym, attempting to prove, that "the disease in question is highly contagious; and, that its mortality may be prevented by the establishment of quarantine and a good police; and that peculiarities belonging to it had been ascertained by the Doctor which were unknown before; namely, that of its attacking the human frame but once:" a discovery so important, so devoutly to be wished, led me to go on with the facts brought in support of so novel a doctrine with all the earnestness they merited.—You are aware, my dear Sir, that, at the time Dr. Pym's book reached me, the establishment at Barbadoes, under my care, was ordered to be broken up, and that I was preparing to return home.

I had, however, previously to any information of this event, transmitted two letters to the Commissioners for Sick and Hurt; the first written to shew the decisively happy effects of the depletory mode of treatment, in the fevers which had attacked the crews of the Charybdis, Ister, and Raven, almost universally: the latter, to shew the deleterious influence of green wood stowed away in contact with bilge water; the decomposing of which, acted most powerfully and fatally on the health of the crew of the Regalia transport. Such causes, in less intensity, I believe to have operated on the crews of the ships before mentioned. The character of the fever on board these several vessels was, in its chief points, perfectly similar; there were marked a very high degree of increased vascular action, of affection of the head, and in many cases, great irritability of stomach accompanying the whole febrile state; but there was not among them, *after their arrival* in Carlisle Bay, one case of coffee-coloured vomiting. Previously thereto, the Charybdis had lost a lieutenant (I believe her first) and two men on her passage from Antigua; in all of which, the above symptom was present at the close of the disease. Alarmed at this commencing mortality, no time was lost in succeeding invasions, in immediately abstracting blood, and sending the cases to the hospital; not another death occurred, although, if my memory serves me, between sixty and seventy were admitted. Were these fevers "the endemic non-contagious of warm climates, arising from miasmata, or of foreign origin, induced with peculiarities distinguishing them from all others, and having the powers of propagation by a specific contagion?"

It has been stated, that, with those who died, black vomiting concluded the scene. The insidious approach of the disease I well recollect to have struck me very forcibly; preceding the high febrile action, there was great depression of muscular power; there were considerable coldness of skin, vertigo, and loose belly; and in considerations founded on this state, blood was not very largely drawn in the first attacks. Indeed, until the action of the cutaneous vessels was restored, by immersing the patient in warm water, the efficacy of the lancet would have been doubtful. But is it not probable, that to its free use, succeeding the effects of the warm water, may be ascribed the exemption from disorganization of the stomach, which took place in those who died?

If black vomiting be a test of the nature of the fever, it may not be too much to assert, that this was what Mr. Pym would have designated a case of "Bulam;" in which the practice of bleeding is strictly prohibited. To which ever of the three classes it may be adjudged, certain it is, that no communication of disease was suspected from one individual to another.

The deep yellowness observed by Mr. P. in the advanced stages of the first variety, is not uncommon where the disease has not been disturbed by the officiousness, or aided by the skill of the physician. However freely may the inverted action of the stomach have been solicited by emetics; with whatever profuseness blood may have been abstracted; if the secretions of the lower belly have not been attended to, and the intestinal canal fully emptied by gentle, yet continued purging—yellowness will be a frequent accompanyment of the latter stage, though, by no means, to be considered a test of its character, being rather the consequence of the mode of treatment pursued. The same remarks are equally applicable to the second variety.—As regards the respect shewn by the third (variety) to natives of a warm climate, or Europeans whose constitutions have been assimilated to a warm climate, by attacking them in a comparatively mild form, you will discover, as you proceed with my letter transmitted to the board, proofs that it occasionally, not only attacks, but fatally, those born in the West Indies. That at Barbadoes, just previously to my quitting it, a reverend gentleman, not more generally known than respected for the even tenour of his life, which had attained nearly fifty years chiefly in that island, was attacked so insidiously, that, but for the non-secretion of urine, and the presence of black vomiting, his attending physician and brother, Dr. Caddell, a man of most excellent judgement, had not suspected the real nature of the malady.

An instance occurred in my own person of a second attack of the disease, each so strikingly marked as not to admit a doubt; you will find others equally as strong in the letter afore-named.

To prove the non-liability of the frame to a second invasion, it was necessary to give the fever a specific character. In this, the facts adduced in my paper will help to shew Mr. Pym has not succeeded. I have referred the commissioners to Mr. Forbes, now deputy-inspector of hospitals in the West Indies, for farther information as to the topography of Fort Royal; but especially Fort Edward, Martinique; wishing rather that the opinion of an old experienced surgeon of the army, so exactly according with my own, should proceed from himself.

Mr. Forbes had the charge of the military at that island for a very considerable time, and will be able to shew, that, whilst in the neighbourhood of the Lamentin Marshes, the inhabitants are very subject to intermittents; so, in like manner, are those more exposed to the exhalations therefrom. That while the people residing about the Careenage enjoy good health, detachments quartered in Fort Edward have suffered most severely from the worst kind of fever, and that the mortality has always been greater when intense heats have succeeded heavy rains.

In reference to such opinions, I will here insert an extract of a letter I wrote to Dr. Dickson, physician to the fleet, in 1808. "I ought to mention, that the Careenage is much more healthy than the outer bay. The crews of the transports, of the merchantmen unloading there, as well as the Port d'Espagne guard-ship and the prison depôts, have been perfectly exempt (with the exception of one case from the latter) from the attacks of the endemial fever; whilst several transports, ordered out into the outer bay, in consequence of the facility with which new rum of the worst quality was got on board, suffered there very much from endemia and intermittents. I would account for the Careenage being more healthy in this way. Most of the ships were anchored considerably within the verge of the neck of land which projects out from the extremity of the Careenage: the current of air being by this somewhat impeded, takes a new course; and, as one point is sufficient to carry it down the bay, I should say, that vessels lying there would have passing over them whatever noxious vapours the low lands at the back and in the neighbourhood of the Lamentin marshes might evolve, and thereby subjecting their crews in a greater degree to attacks of disease."

This extract may appear the less irrelevant, especially when it is meant as an introduction to my report of the fever at Mariegalante, and may apply equally to Mr. Pym, in his first acquaintance with that at Fort Edward; offering, if not in result an equal calamity, at least a malignity of character very conspicuous. The conclusions drawn by us are very dissimilar; Mr. Pym ascribing to contagion what, I think, may have been more truly attributable to miasmata, as was the case at the former place. Had the fever pervading the 70th regiment been of an infectious nature, we can scarcely reconcile its immediate disappearance on their removal to Point Negro; it is contrary to every law obtaining in other infectious disorders. But the change of situations, if miasma be admitted as the cause (and the first appearance, as well as progress, are strongly corroborative of the opinion, that to it should it be ascribed), is of itself sufficient to account for the benefits so suddenly effected. Fort Edward is surrounded by deep moats, many of them full of stagnant water, having the effluvia from the marshes and canal in its rear constantly passing over it, whilst there is not a marsh, nor an unhealthy spot, within any reasonable distance of either Point Negro or Cape Pilot. Mr. Pym's own attack was, in all probability, the consequence of unceasing activity in the prosecution of his professional pursuits, which must have often led him to, and detained him long at, the fort. There is much accordance with the effects of the atmosphere upon the settled inhabitants at Martinique as described by the author, and that given by me as experienced by the people of Mariegalante. They also had their slice of lemon sprinkled with salt to their temples, and their ptysans of imperial, remedies common to West Indians, whether French or English.

The decline as well as appearance of epidemics depends so much on the importation of new comers, that a true judgement cannot well be formed of the greater or less salubrity of any part of the world, without a better acquaintance with that circumstance than authors often afford us. Thus, for example, it is stated by Mr. Pym, that a part of the coast of Spain continued free of disease for three years, when fever became epidemic. Had Mr. P. at the same time, informed his readers what number of troops had been withdrawn and replaced by others, or whether there had been any other change in the population, his book would have been more valuable.

From 1811 to 1816, the West India reports of health presented comparatively fewer attacks, and less fatality, than for any preceding equal number of years; but it should not be forgotten, that, during these, there were few importations of unseasoned subjects: and it may not be amiss to recommend an inquiry into the number of attacks and issues of disease among those employed in the land and sea service, who have been sent out on the peace establishment, to relieve the seasoned veterans of that climate; particularizing those who may have recently served there in the war.

In proceeding with Mr. Pym's publication, the occurrences on board the four transports would seem to have a great similarity with those in the Regalia, the history of which I have given you; Dr. P. asks Dr. Bancroft, where was the marsh producing these attacks in the four ships? Without anticipating the Doctor's reply, this I am assured of, that the minds of medical men, just arriving in the West Indies, have often been needlessly tormented in accounting for the production of fever, where no marshes or still water were to be found.

All the varieties of miasmata we are not acquainted with; but we possess enough of information to know, that fevers are often produced by others than those of marshes, and those of a much higher degree of intensity and fatality.

The appearance and propagation of a disease, possessing in itself a specific character, depend on causes generally every where alike: these would not seem to have obtained in the state of the weather at Gibraltar, as described by Dr. Nooth, "to have been and to continue excessively dry and hot;" whilst at Jamaica, where the 54th suffered much, Dr. Rocket ascribes its appearance "to the heavy and incessant rains for the last fortnight."

The fact mentioned in my letter of the Pompée's ship company being seized with fever, in the removal of the prisoners from the French brig Palineure, may be considered evidence towards proof, that the sources of disease are capable of transportation in baggage. Should it be so admitted, the difficulty of accounting for the fever which was common in the detachment of the 54th at once ceases. Something not unlike this occurred among the marines on board the Dragon, when Sir F. Laforey had his flag in her. There had not been any attacks on board for a considerable time, when suddenly one, two, and several other marines were seized in quick succession; my recollection does not serve me at this distance of time, how many were invaded, but I believe the greater number attacked, died. The ship went to sea; the disease as suddenly ceased, *not a sailor was attacked*: Was this a contagious fever communicable from one individual to another? Why was it that the marine corps were exclusively seized? and why should it cease so suddenly, on the ship going to sea? I have thought these worthy of mention, as assisting to shew how inexplicable are sometimes the rise and cessation of epidemics. Should a correct history of this be called for, it may be found on the records of the Transport Office, as noted by my esteemed friend, Doctor Creighton, then surgeon of the ship.

At page 133, I find proofs of exemption from any fatal epidemic, among men stationed in the Careenage, Fort Royal, Martinique, thus corroborated by Doctor Gillespie, whose long residence in the West Indies, and extensive means of observation as surgeon of the Naval Hospital there, give to him claims for the correctness of his remarks. "During the three last months of 1795, and the three first of 1796, whilst the garrisons of St. Pierre and Fort Royal (that is, Fort Edward) were still more fatally preyed on by the malignant epidemic, which I have described as affecting the seamen; the prisoners of war crowded half naked into badly aired transports (in the Careenage) remained free from any fatal epidemic."

Much other matter occurs to me in the perusal of Mr. Pym's book, which I should notice farther, were it not, that the little access I have of late had to the recent publications on fever inclines me to think, I should be anticipated by men who have had extensive means of forming a true judgement of the weight that ought to attach to the novel doctrines of the author.

I am, dear Sir,

Yours most truly,

JOHN MORTIMER.*

* Mr. Mortimer's Official Report will appear in our next Number. Ed.

